# Supplementary figures and images for: Role of the Mitochondrial Citrate-malate Shuttle in Hras12V-Induced Hepatocarcinogenesis: A Metabolomics-Based Analysis
Source: Metabolites. 2020 May 13;10(5):193. doi: 10.3390/metabo10050193 (PMC7281175; doi:10.3390/metabo10050193)

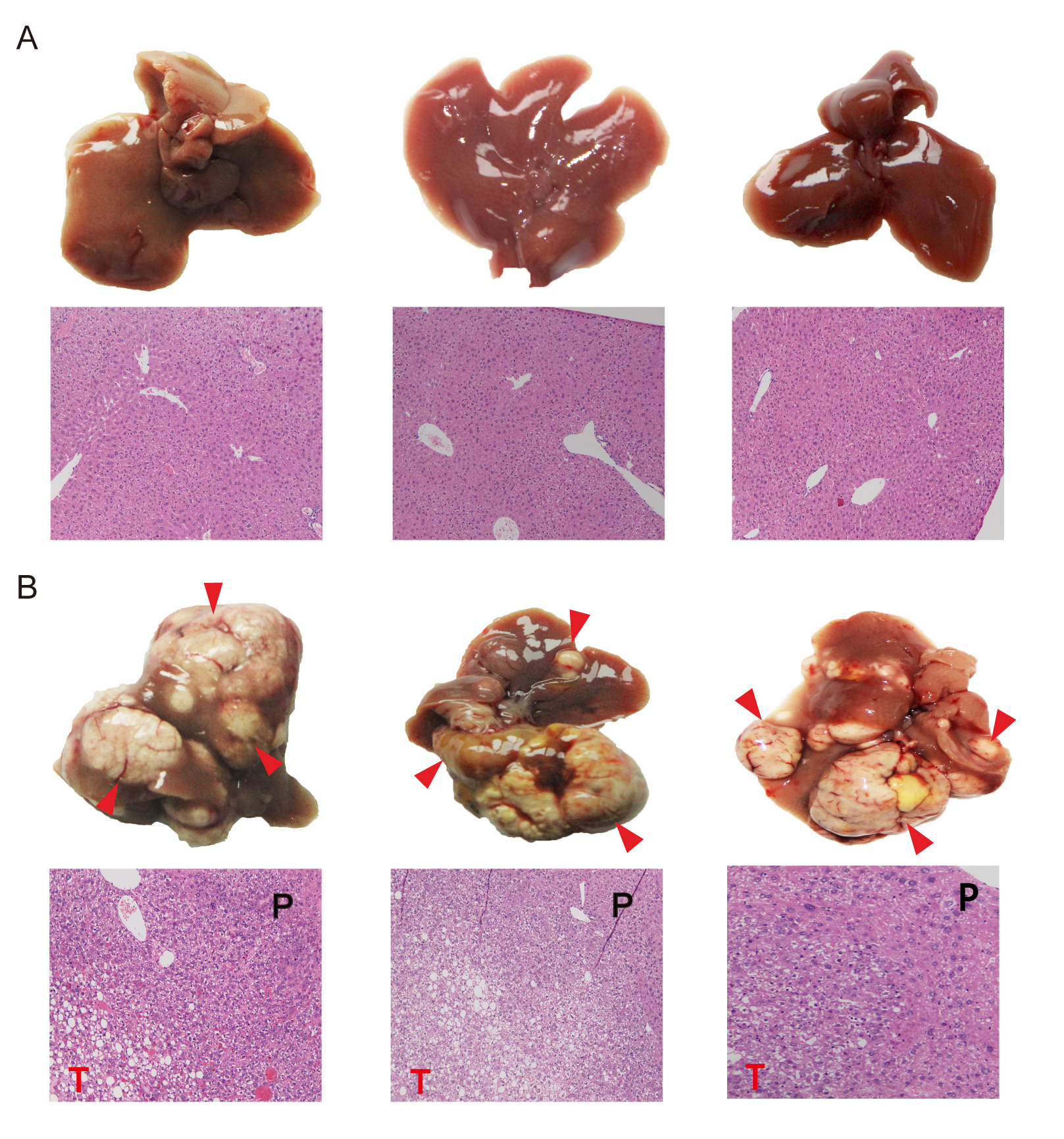

Supplement: Supplementary file 1 [file metabolites-10-00193-s001.zip › supplementary materials/Figure S1. Anatomic and histopathological analysis of hepatic alterations..tif]

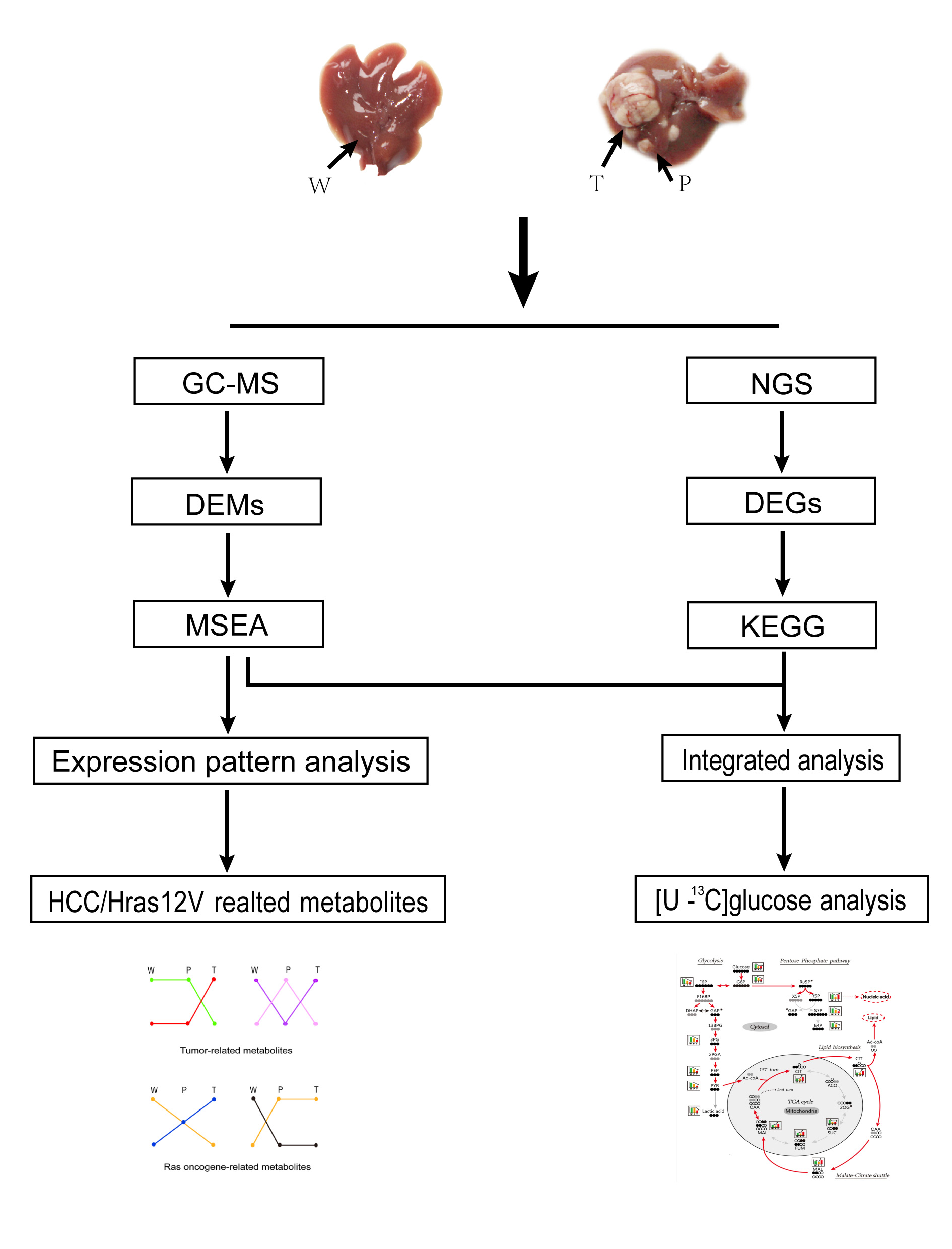

Supplement: Supplementary file 1 [file metabolites-10-00193-s001.zip › supplementary materials/Figure S2. Experimental design.jpg]

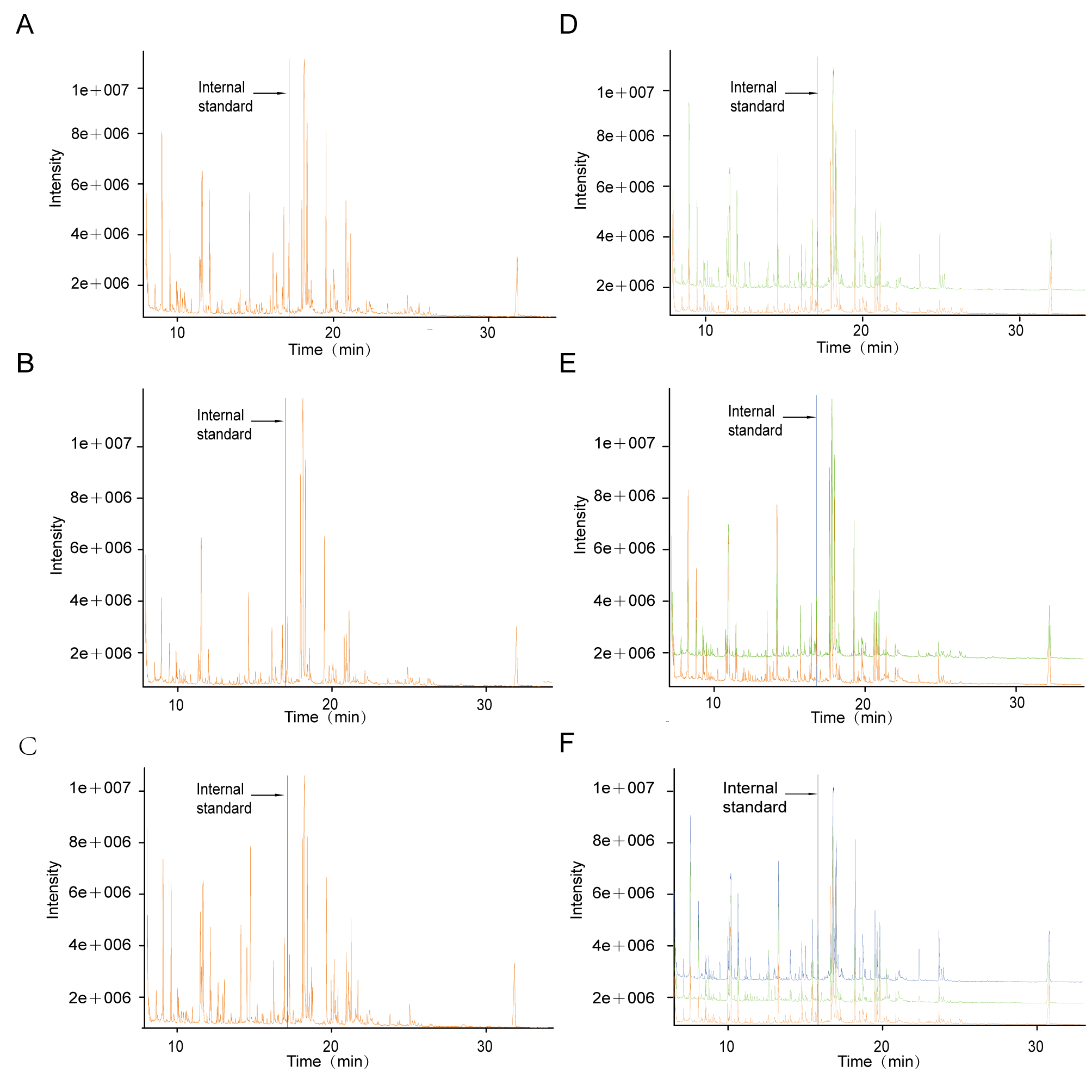

Supplement: Supplementary file 1 [file metabolites-10-00193-s001.zip › supplementary materials/Figure S3. Typical GCMS total ion current (TIC) chromatograms.tif]

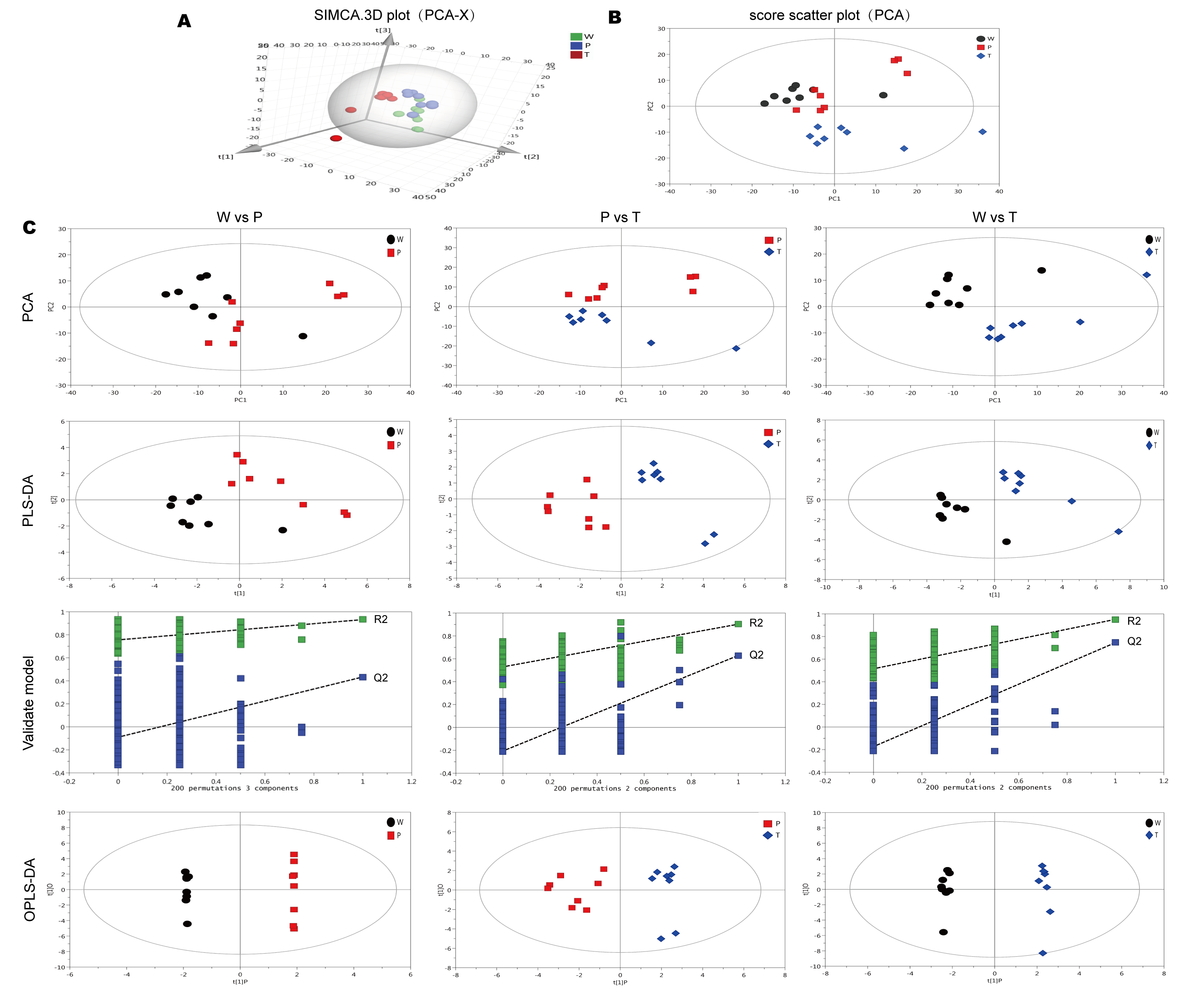

Supplement: Supplementary file 1 [file metabolites-10-00193-s001.zip › supplementary materials/Figure S4. Score plots of PCA, PLS-DA, and OPLS-DA based on the metabolite profile data.tif]

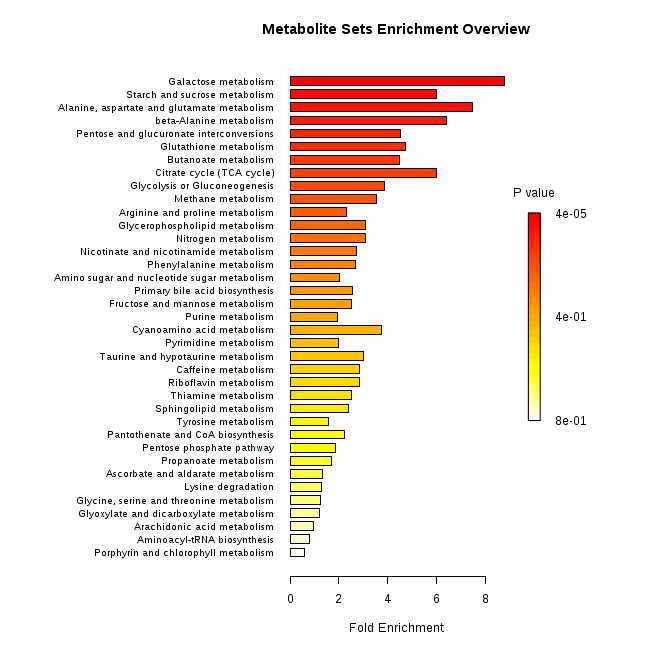

Supplement: Supplementary file 1 [file metabolites-10-00193-s001.zip › supplementary materials/Figure S5-A. Pathways enrichment analysis for significantly changed metabolites between W and P.png]

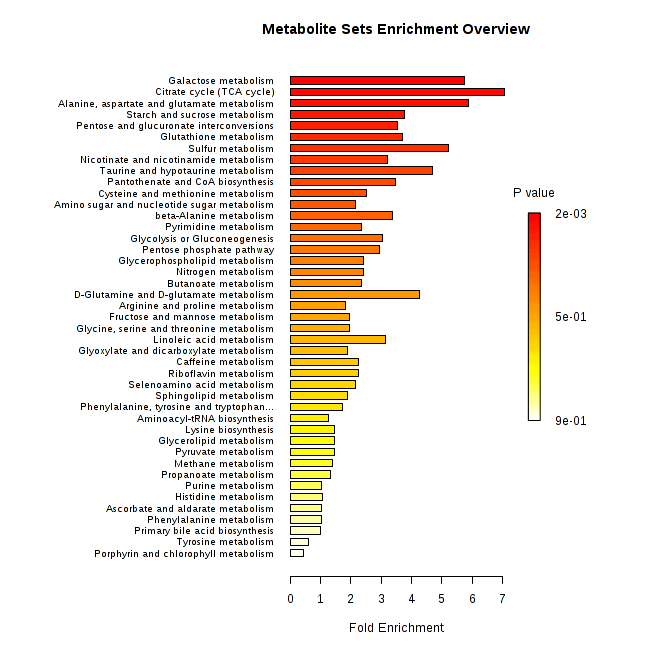

Supplement: Supplementary file 1 [file metabolites-10-00193-s001.zip › supplementary materials/Figure S5-B. Pathways enrichment analysis for significantly changed metabolites between P and T.png]

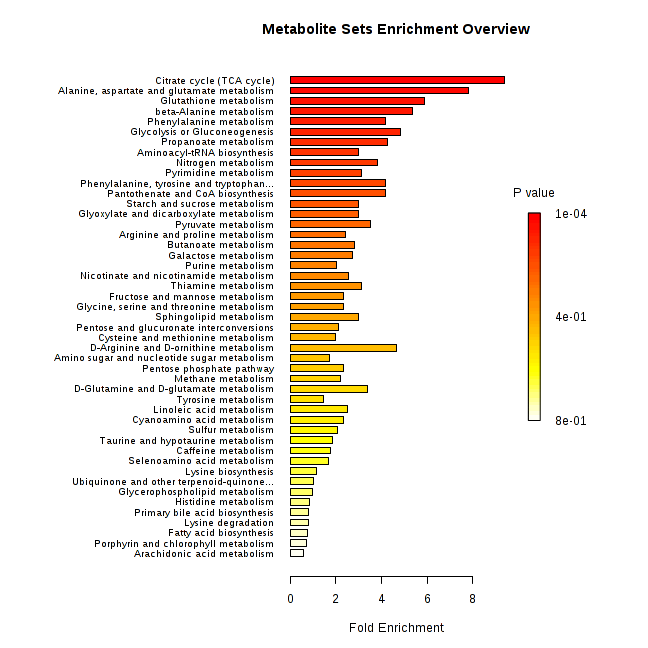

Supplement: Supplementary file 1 [file metabolites-10-00193-s001.zip › supplementary materials/Figure S5-C. Pathways enrichment analysis for significantly changed metabolites between W and T.png]

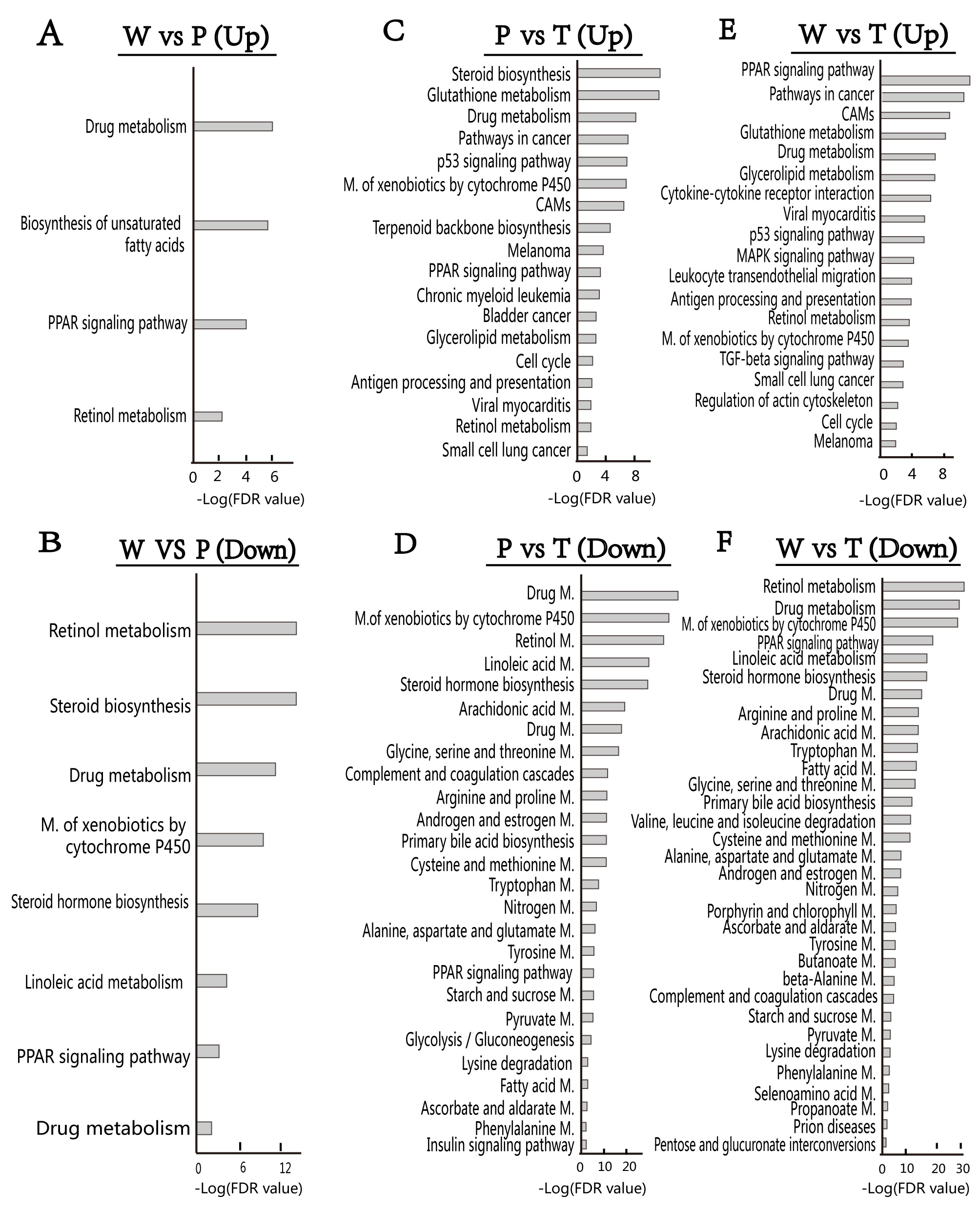

Supplement: Supplementary file 1 [file metabolites-10-00193-s001.zip › supplementary materials/Figure S6. KEGG pathway enrichment analysis for significantly changed genes.jpg]

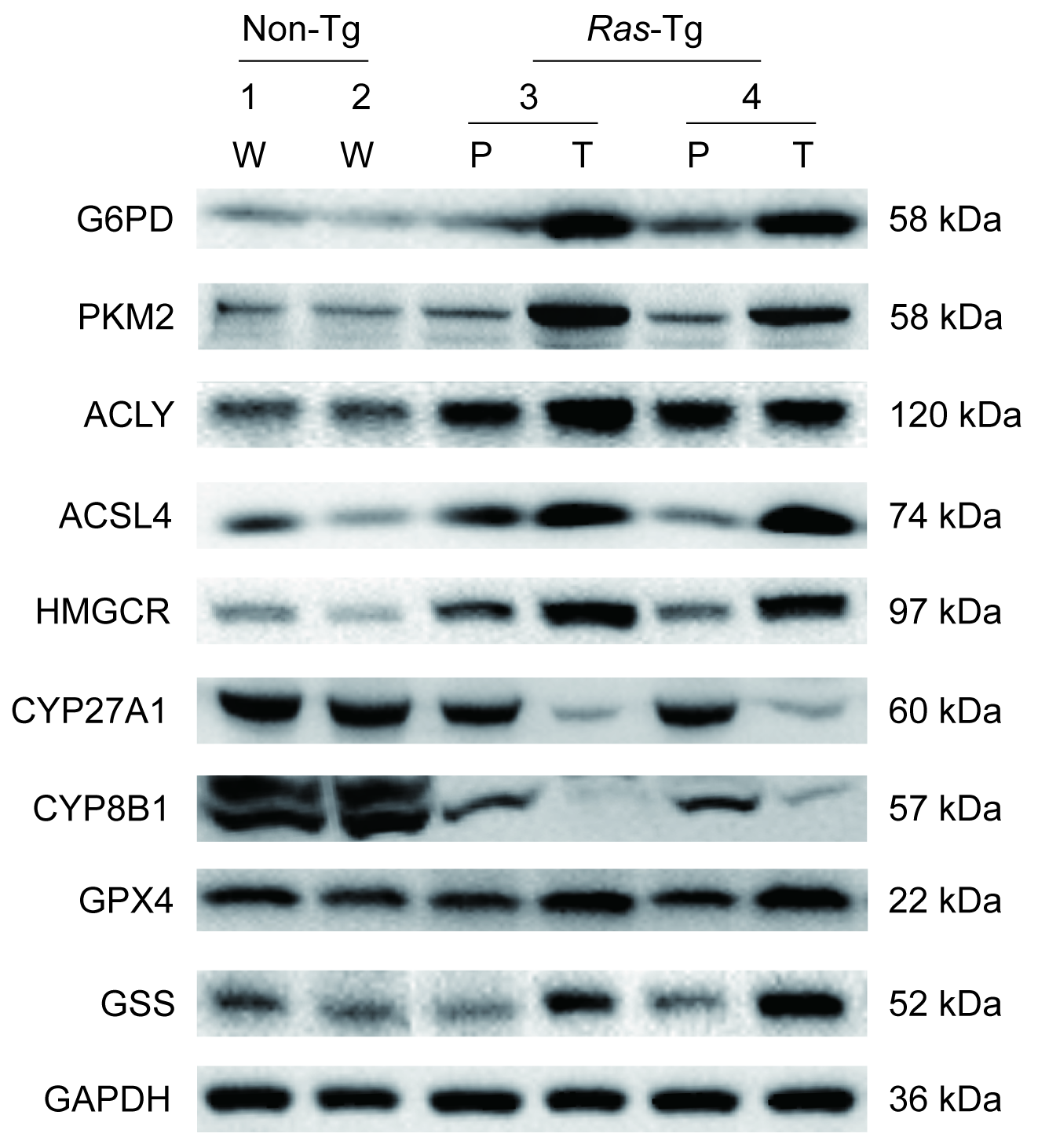

Supplement: Supplementary file 1 [file metabolites-10-00193-s001.zip › supplementary materials/Figure S7. Validation of DEGs in HCC, precancerous and normal liver tissues by Western blot.tif]

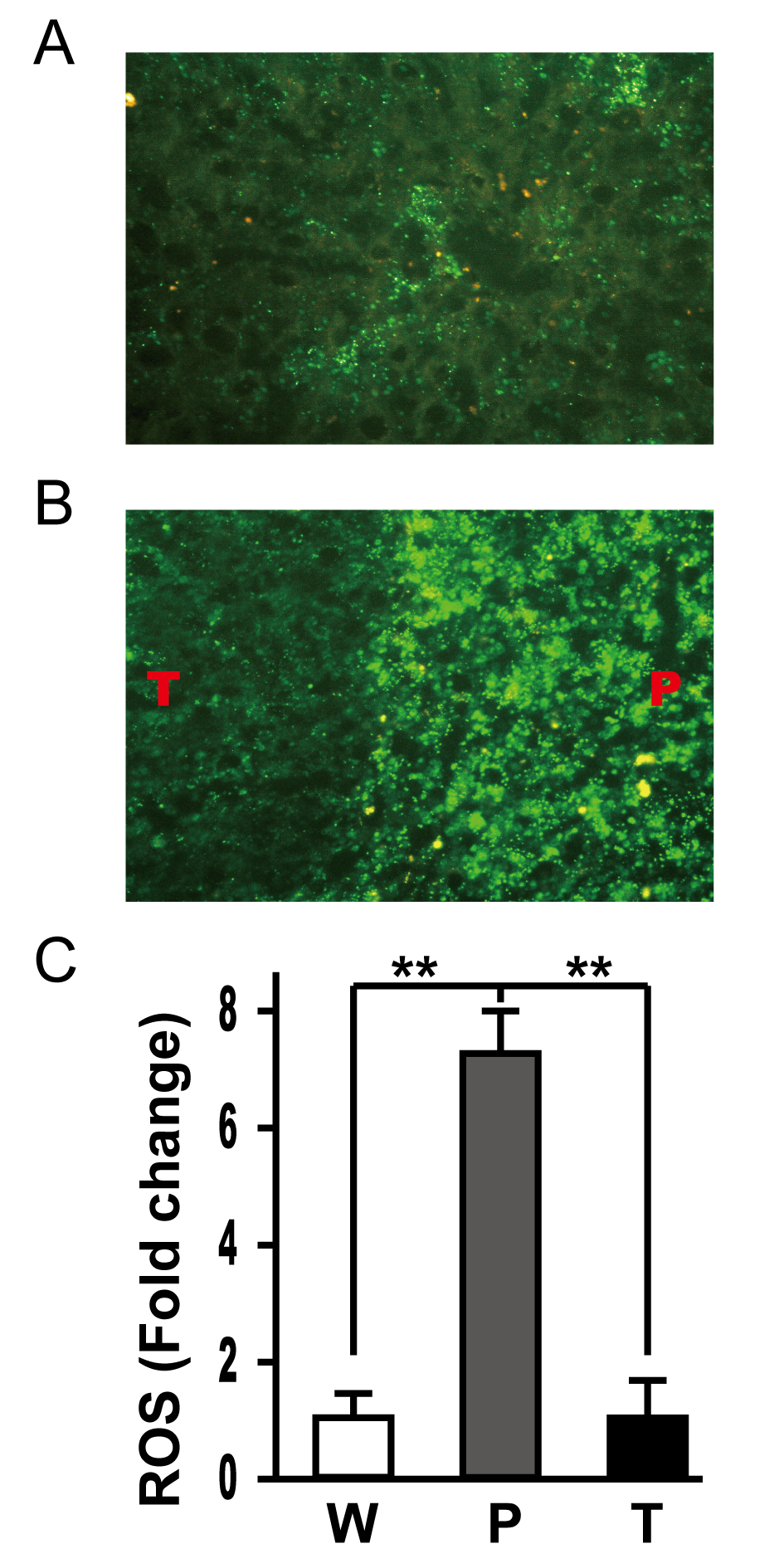

Supplement: Supplementary file 1 [file metabolites-10-00193-s001.zip › supplementary materials/Figure S8. The tissue ROS levels detected by fluorescent assay.tif]

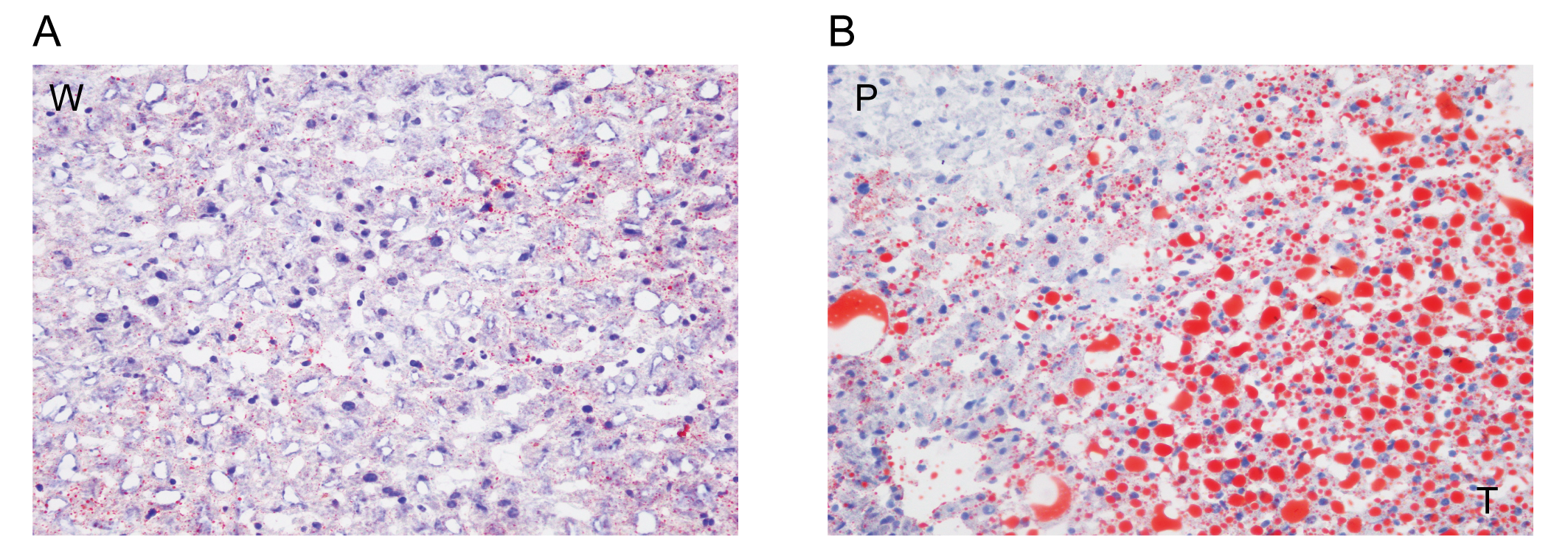

Supplement: Supplementary file 1 [file metabolites-10-00193-s001.zip › supplementary materials/Figure S9. Tissue stained with Oil Red O.tif]
