# Supplementary material for: Role of the Mitochondrial Citrate-malate Shuttle in Hras12V-Induced Hepatocarcinogenesis: A Metabolomics-Based Analysis
Source: Metabolites. 2020 May 13;10(5):193. doi: 10.3390/metabo10050193 (PMC7281175; doi:10.3390/metabo10050193)
Supplement: Supplementary file 1 [file metabolites-10-00193-s001.zip › supplementary materials/Supplementary Information.doc]

**Supplemental Information**

**Materials and Methods**

**1. Histopathological diagnoses**

Tissue samples were fixed in 10% neutral buffered formalin, embedded in paraffin, sectioned, and stained with hematoxylin/eosin according to standard methods. The histopathological diagnoses were based on the criteria described by Frith and Ward . The normal liver tissues of C57BL/6J wild-type non-transgenic males showed the normal architecture and cells with granulated cytoplasm and small uniform nuclei. The Hras12V transgenic (Ras-Tg) males developed multiply hepatic tumors including hepatocellular adenoma (HCA) and hepatocellular carcinoma (HCC) with high reproducibility. For 9 month-age old Ras-Tg males, the incidence for HCA is 94.1% and for HCC is 88.2% . The morphological changes of hepatic tumors seen in the Ras-Tg are listed below. Gross findings for hepatic tumors were a yellowish-brown color, and were of various sizes with single or multiple nodules in the liver. Microscopic hepatic HCA were relatively well-circumscribed and the hepatocytes around the tumor lesions were compressed. HCA was composed of hepatocytes with excess fat vacuoles, varying size, and cellular atypia such as pleomorphic nuclei and sinusoid compression, and did not involve central veins or portal triads. Relative to HCA, the HCC was: (1) large in size, and typically more than 5 mm in diameter; (2) showed a trabecular arrangement of tumor cells; and (3) contained highly anaplastic cells with evidence of necrosis.

The precancerous tissues are the morphological normal liver tissues between hepatic tumors, in which the hepatocarcinogenesis is induced by hepatocyte-specifically expressed Hras12V oncogene. Microscopic findings of the precancerous liver tissues showed largely the same as normal liver tissues, but with scattered distribution of histopathological changes including fat vacuoles, necrosis, edema, and altered hepatic foci surrounding the central vein of hepatic lobule. The altered foci included the aggregation of atypical hepatocytes with basophilic cytoplasm and hyper-chromatic nuclei. As the hepatic tumors were protruded from the precancerous tissues and had a clear boundary between them. It was easy to accurately sample and had a slim chance to mix the hepatic tumor tissues with the precancerous tissues.

**2. Nontargeted-metabolomics analysis**

***Sample preparation for GC-MS analysis***

The histopathologically identified tissues (eight individual samples for each group) were subjected to two derivatization steps for GC-MS analysis. Approximately 0.05 mg of each sample was collected in 2-mL EP tubes and extracted with 0.4 mL methanol–chloroform (V methanol: V chloroform = 3:1). Then, 20 *μ*L of L-2-chlorophenylalanine (1 mg/mL stock in dH2O) was added as an internal standard, and the sample was homogenized in a ball mill for 3 min at 65 Hz. After the samples were centrifuged, the supernatant was transferred to a 2-mL GC-MS glass vial. An equal volume of 14 *μ*L was taken from each sample and placed into the 2-mL GC-MS glass vial as a mixed sample for quality control. The extracts were dried in a vacuum concentrator without heating at 37°C for approximately 2 h. Then, 80 *μ*L of methoxylamine hydrochloride (20 mg/mL) was added to the dried metabolites and incubated at 80°C for 20 min in an oven after mixing and sealing. The lid was opened, and 100 *μ*L of Bis (trimethylsilyl) trifluoroacetamide (BSTFA) (containing 1% trimethylchlorosilane (TMCS), v/v) was added to each sample, which was sealed again and incubated at 70°C for an hour. Then, 5 *μ*L of FAMEs (a standard mixture of fatty acid methyl esters, C8-C16: 1 mg/mL; C18-C24: 0.5 mg/mL in chloroform) was added to the mixed sample, cooled to room temperature, and mixed well for GC-MS analysis.

***GC-TOF-MS analysis***

GC-TOF-MS analysis was performed using an Agilent 7890 gas chromatograph system coupled with a Pegasus HT time-of-flight mass spectrometer. The system utilized an Rxi-5Sil MS column (30 m × 250 *μ*m inner diameter, 0.25 *μ*m film thickness; Restek, USA). A 1-*μ*L aliquot of the analyte was injected in splitless mode. Helium was used as the carrier gas, the front inlet purge flow was 3 mL/min, and the gas flow rate through the column was 20 mL/min. The initial temperature was 50°C for 1 min, and then it was increased to 330°C at a rate of 10°C/min, and then maintained for 5 min at 330°C. The injection, transfer line, and ion source temperatures were 280°C, 280°C, and 250°C, respectively. The energy was −70 eV in electron impact mode. The mass spectrometry data were acquired in full-scan mode with an m/z range 30–600 at a rate of 20 spectra per second after a solvent delay of 366 sec.

***Analysis of GC-MS data***

First, the chroma TOF4.3X software of the LECO Corporation and LECO-Fiehn Rtx5 database were used for the extraction of raw peaks, the filtering of data baselines, and the calibration of the baseline, peak alignment, deconvolution analysis, peak identification, and integration of the peak area. The RI (retention time index) method was used in peak identification, and the RI tolerance was 5000. Then, identified GC-MS data were imported into LineUp (Infometrix, Bothell, WA) and PiroTrans (GL Science) to align the chromatograms based on the peak intensity and the retention time of the internal standard, 2-hydroxyundecanoic acid. The generated peak lists were imported into Pirouette software (Infometrix, Woodinville, WA) for multivariate statistical analysis. The following composition analysis of the samples was performed using MetaboAnalyst version 3.0 (http://www.metaboanalyst.ca/). The analysis methods included Principal Component Analysis (PCA), Partial Least Squares-Discriminant Analysis (PLS-DA), Orthogonal Projections to Latent Structures-Discriminant Analysis (OPLS-DA), and model validation.

***Metabolite set enrichment***

Metabolite set enrichment was investigated using metabolite set enrichment analysis (MSEA) and the overrepresentation analysis (ORA) tools offered by MetaboAnalyst version 3.0. Briefly, all metabolites from the GS-MS array dataset were entered into MSEA as a list of metabolites. The mouse pathway library was chosen, and the ORA algorithm was selected using a hypergeometric test. The pathway ORA tool then used the proportion of differentially expressed metabolites in the whole array to determine whether a particular pathway was significant. A list of pathways associated with the uploaded metabolites was produced. *P* < 0.05 was considered to represent significant differences.

**3. Transcriptomics analysis**

***Different gene expression analysis by NGS***

Different gene expression was detected using NGS analysis. Tissue samples of W, P, and T (5 for each group) were chosen for total RNA extraction using TRIzol reagent (Invitrogen, Grand Island, NY). Due to the consistent inbred genetic background, the definite etiology (*ras* oncogene) for HCC development, and the consistent pathological features of the samples in each group, the total RNA samples from the same group were mixed equivalently to generate two composite samples, that is, HCC and wild-type liver tissues. Preparation of the mRNA sample for RNA-Seq analysis was performed using the TruSeq RNA LT Sample Prep Kit v2 (Illumina, San Diego, CA). The template molecules were used for cluster generation and sequencing on an Illumina HiSeq 2000 (Illumina, San Diego, CA) instrument. One sample per lane was used to generate 100-bp paired-end reads. Read alignment to the UCSC Mouse Reference Genome (mm10, http://genome.ucsc.edu/) was performed using Tophat v1.3.2. Transcript assembly and quantification were performed by Cufflinks v2.0.2 to process the Tophat alignments. The assembled Cufflinks transcripts were processed with Cuffcompare analytical strategies to detect differentially expressed genes. The database for annotation, visualization, and integrated discovery (DAVID) v6.7 was used to interpret the differentially expressed gene pools and KEGG pathway enrichment assay.

**4. Targeted-metabolomics analysis with [U-^13^C] glucose**

***[U-^13^C] glucose injections and tissue extraction***

Nine month-old *Ras*-Tg and C57BL/6J wild-type males were fast overnight with ad lib provided water. [U-^13^C] glucose was dissolved in PBS, aseptically filtered, and then tail-intravenous injected at a single dose of 750 mg/kg. Forty-five minutes later, liver tissue was perfused with ice cold PBS and W, P, and T tissues were immediately harvested and flash-frozen in liquid nitrogen. The mouse liver samples (40±1 mg) were homogenized in 20 volumes (vol/wt) of methanol/water (1:1, v/v) solution with TissueLyser JX-24 (Jinxin, Shanghai, China) with beads at 30 Hz for 3 min. The homogenates were vortexed for 1 min before centrifugation at 4 °C and 14000 g for 15 min. Totally, 500 μL supernatant was evaporated to dryness under nitrogen gas. The residue was reconstituted in 100 μL of 50% aqueous acetonitrile, and after centrifugation the supernatant was performed in ultra-performance liquid chromatography-tandem mass spectrometry (UPLC-MS/MS) analysis .

## *UPLC-MS/MS Analysis of BCKAs*

UPLC-MS/MS analysis was performed on a Waters Acquity UPLC system (Waters, Milford, MA) coupled to a Triple Quad™ 5500 tandem mass spectrometer (AB Sciex, Framingham, MA). The samples were separated using a Waters UPLC BEH Amide column (100 mm× 2.1 mm, 1.7 μm) with a linear gradient elution system of mobile-phase (A) 10 mM ammonium acetate and 0.1% ammonium hydroxide in water and (B) 10 mM ammonium acetate and 0.1% ammonium hydroxide in acetonitrile/water (90/10, v/v). The gradient elution conditions were 0-1 min, 95% B; 2 min, 70% B; 6 min, 60% B; 6.1 min, 50% B; 7 min, 50% B; 7.1 min, 95% B; 12 min, 95% B. The column temperature was 30℃. The injection volume was 5 μL. The flow rate was maintained at 300 μL/min.

The analytes separated from column were ionized in an electro spray ionization source in negative mode (ESI-). Source temperature: 500℃, curtain gas (CUR): 25 psi, ion source gas 1(GAS1): 50 psi, ion source gas 2 (GAS2): 50 psi, collision gas (CAD): 8 psi, ion spray voltage (IS): -4500V, entrance potential (EP): -10 V, collision cell exit potential (CXP1): -10V. The dwelling time was set at 20 ms. The multiple reaction monitoring (MRM) was used to acquire data in optimized MRM transition (precursor > product), declustering potential (DP), and collision energy (CE) as Table S7. AB Sciex Analyst software (version 1.5.2) was used to control instruments and acquire data.

## *Mass spectrometry data pretreatment*

Analyst (version 1.5.2, AB Sciex) was used to analyze data. Using the default parameters and assisting manual inspection to ensure the qualitative and quantitative accuracy of each compound, extract and output chromatographic retention time and peak area. The intensities of the labeled metabolites were presented as the ratio of the peak area of the labeled metabolite to the total peak area pool of that metabolite (labeled/total).

**5. Metabolite detection**

*NADPH:* The level of NADPH was detected using a NADP/NADPH Quantification Colorimetric Kit (Cat No. k347-100; BioVision, Milpitas, USA) according to the instructions of the manufacturer. Briefly, the frozen sample (~50 mg) was washed with ice-cold 1 × PBS and homogenized in 0.5 mL of NADPH extraction buffer. After keeping the samples on ice for 10 min, the homogenate supernatant was obtained by centrifugation. Two-hundred microliters of supernatant was transferred into an Eppendorf tube and heated at 60°C for 30 min in a water bath. Then, a 50-*μ*L aliquot was added to the labeled 96-well plate in duplicate, and the NADPH level was detected by following the instructions for the kit.

*Triglyceride and cholesterol:* The frozen samples (~50 mg) were used for lipid extraction. Each frozen tissue was added to a 10-mL heptane–isopropanol–tween mixture (3:2:0.01 by volume) and homogenized. This homogenate was centrifuged, and the supernatants were collected and evaporated with nitrogen flow. The triglyceride and cholesterol contents were determined using commercial kits (Cat No. A0-10017 and A0-10027; Dongou Biological Engineering Co Ltd, Wenzhou, China). All samples were measured in duplicate.

*Pyruvate, lactate, and bile acid:* The frozen samples (~50 mg) were added to 0.5 mL of 1 × PBS (0°C, pH 7.2–7.4) and homogenized on ice. The homogenate supernatant was obtained by centrifugation, and the levels of pyruvate, lactate, and bile acid were detected using commercial kits according to the instructions of the manufacturer (Cat No. A081, A019-2, and E003-2; Nanjing Jiancheng Bioengineering Institute, Nanjing, China). All samples were measured in duplicate.

*GSH：*Fresh samples (~50 mg) were added to 1 mL of ice-cold 1 × PBS and homogenized with a glass homogenizer on ice. The resulting suspension was sonicated with an ultrasonic cell disrupter until the solution was clarified. Then, the homogenates were centrifuged for 5 min at 10,000×*g*. The supernatant was collected and detected immediately using an enzyme-linked immunosorbent assay kit according to the instructions of the manufacturer (Cat No. CEA294Ge; Cloud-Clone Corp, Wuhan, China). All samples were measured in duplicate.

*ROS：*The mouse reactive oxygen species (ROS) ELISA Kit (BH8443, BOYAO Biotechnology, Shanghai, China) was used to detect the ROS levels, and the procedures were performed according to the instructions of the manufacturer. Briefly, the fresh tissue samples were cut, weighed, and placed into a grinding tube containing ceramic beads and 1 mL of pH 7.4 PBS. Then, the tissue was homogenized and centrifuged, and the supernatant was carefully collected and assayed with an ELISA Kit. The final ROS level was calculated by dividing the total ROS content by the original tissue weight.

**6. Quantitative Real-time PCR (RT-qPCR) validation**

To confirm the results of transcriptome sequencing analysis, the relative mRNA expression levels of 21 randomly selected genes in RNA from another sample set of W, P and T tissues of male and female were evaluated. Total RNA of samples was isolated using the TRIzol reagent (Tiangen Biotech, Beijing, China). According to manufacturer’s instructions, three micrograms of total RNA were reverse transcribed to single-stranded cDNA using a reverse transcription system (Tiangen Biotech, Beijing, China) and then performed qPCR experiments using Real Master Mix SYBR Green (Tiangen Biotech, Beijing, China) on StepOne Plus^TM^ Real-Time PCR System (Applied Biosystems). The primers used to detect the expression levels of target genes were listed in Table S6. A separate RT-qPCR using primers for the detection of *Rp135a* was used as a control. All PCR experiments were performed in duplicate and the expression levels of genes were calculated against the *Rp135a*.

**7. Western blot analysis**

Liver issues were homogenized on ice in 10 mM Tris buffer (pH 7.4) with 1 mM EDTA and Roche complete protease inhibitor mixture (PhosSTOP EASYpack, REF: 4906845001), centrifuged at 10,000 g for 30 min and then the concentration was determined by Bradford assay (BioRad). Protein samples were separated on 12% polyacrylamide gels and transferred to PVDF membranes (Amersham Biosciences, Uppsala, Sweden). After blocking with 5% skim milk, primary and secondary antibodies were used to incubate the members. Immunoreactive complexes were visualized using ECL reagents (Biotool, Shanghai, China). The detailed information of the antibodies used in the present study is list in Table S8.

**8. Oil red O staining**

For oil red O staining, 8 μm thick frozen liver sections were fixed in 4% paraformaldehyde for 10 min, washed with deionized water 3 times and stained with oil red O working solution (Solarbio, G1260) for 10 min at room temperature. After washing with deionized water and stained with hematoxylin, the slides were examined under optical microscope.

**9. Detection of ROS in liver tissue by fluorescence immunoassay**

The level of ROS in liver tissues was detected by fluorescence immunoassay according to the instructions of the kit (GMS10016; GENMEG Scientifics INC, Shanghai, China). A 5 μ m frozen section was made from the fresh mice liver tissue. The section was incubated with GENMED cleaning solution A at room temperature for 5 min, and then incubated with GENMED staining solution at 37°C for 1 h in dark. After washed with PBS in dark for 5 min, anti-fluorescence quench sealer was added. Finally, under the 490nm excitation light and 520nm scattered light, the photos were taken by fluorescence microscope. Image Proplus 6.0 (IPP) image processing software was used to analyze the optical density of fluorescence and calculate the ROS level.

**10. Statistical analysis**

The peak intensities of metabolites and the signal values of gene expression were statistically analyzed by a Student’s *t*-test. All the data were presented as the mean ± SEM, with a level of probability of 0.05 as the criterion for significance. Fold change is a measure describing how much a quantity changes going from the initial to the final value.

.

**Reference:**

1.	Frith CH, Ward JM: **A morphologic classification of proliferative and neoplastic hepatic lesions in mice**. *J Environ Pathol Toxicol* 1979, **3**(1-2):329-351.

2.	Wang AG, Moon HB, Lee MR, Hwang CY, Kwon KS, Yu SL, Kim YS, Kim M, Kim JM, Kim SK *et al*: **Gender-dependent hepatic alterations in H-ras12V transgenic mice**. *J Hepatol* 2005, **43**(5):836-844.

3.	McDonald TS, Carrasco-Pozo C, Hodson MP, Borges K: **Alterations in Cytosolic and Mitochondrial [U-(13)C]Glucose Metabolism in a Chronic Epilepsy Mouse Model**. *eNeuro* 2017, **4**(1).
