# Supplementary material for: Role of the Mitochondrial Citrate-malate Shuttle in Hras12V-Induced Hepatocarcinogenesis: A Metabolomics-Based Analysis
Source: Metabolites. 2020 May 13;10(5):193. doi: 10.3390/metabo10050193 (PMC7281175; doi:10.3390/metabo10050193)
Supplement: Supplementary file 1 [file metabolites-10-00193-s001.zip › supplementary materials/Table S7. Parameters for LC-MS-MS analysis.doc]

| **Compounds** | **Transition (m/z)** | **DP (V)** | **CE (eV)** | **Retention Time**  **(min)** |
| --- | --- | --- | --- | --- |
| G6P-m0, …, m6 | 259>97, …, 265>97 | -50 | -22 | 7.21 |
| F6P-m0, …, m6 | 259>97, …, 265>97 | -50 | -22 | 6.97 |
| R5P-m0, …, m5 | 229>97, …, 234>97 | -80 | -19 | 6.9 |
| Ru5P-m0, …, m5 | 229>97, …, 234>97 | -80 | -19 | 6.79 |
| E4P-m0, …, m4 | 199>97, …, 203>97 | -50 | -14 | 6.23 |
| PEP-m0, …, m3 | 167>79, …, 170>79 | -50 | -19 | 7.02 |
| GAP-m0, …, m3 | 169>79, …, 172>79 | -50 | -14 | 6.7 |
| 2PGA/3PGA-m0, …, m3 | 185>79, …, 188>79 | -40 | -26 | 5.11 |
| S7P-m0, …, m7 | 289>97, …, 296>97 | -80 | -20 | 7.07 |
| glucose-m0, …, m6 | 239>179, …, 245>185 | -50 | -11 | 5.11 |
| 6PGA-m0, …, m6 | 275>97, …, 281>97 | -80 | -20 | 7.38 |

**Table S7.** Parameters for LC-MS/MS analysis
